# Pointing in cervical dystonia patients

**DOI:** 10.3389/fnsys.2023.1306387

**Published:** 2023-11-28

**Authors:** Maria Paola Tramonti Fantozzi, Roberta Benedetti, Alessandra Crecchi, Lucia Briscese, Paolo Andre, Pieranna Arrighi, Luca Bonfiglio, Maria Chiara Carboncini, Luca Bruschini, Paolo Bongioanni, Ugo Faraguna, Diego Manzoni

**Affiliations:** ^1^Department of Translational Research and of New Surgical and Medical Technologies, University of Pisa, Pisa, Italy; ^2^Residency School in Physical Medicine and Rehabilitation, University of Pisa, Pisa, Italy; ^3^Functional Rehabilitation Unit, North-West Tuscany Sanitary Service, Pisa, Italy; ^4^Department of Medicine, Surgery and Neuroscience, University of Siena, Siena, Italy; ^5^Severe Acquired Brain Injuries Section, Integrated Department of Medical Specialties, Pisa University Hospital, Pisa, Italy; ^6^Developmental Neurorehabilitation Section, Maternal and Child Department, Pisa University Hospital, Pisa, Italy; ^7^Department of Surgical, Medical, Molecular Pathology and Critical Cares, University of Pisa, Pisa, Italy; ^8^Department of Developmental Neuroscience, IRCCS Fondazione Stella Maris, Pisa, Italy

**Keywords:** cervical dystonia, neck input, pointing errors, asymmetry, space representation

## Abstract

**Introduction:**

The normal hemispheric balance can be altered by the asymmetric sensorimotor signal elicited by Cervical Dystonia (CD), leading to motor and cognitive deficits.

**Methods:**

Directional errors, peak velocities, movement and reaction times of pointing towards out-of-reach targets in the horizontal plane were analysed in 18 CD patients and in 11 aged-matched healthy controls.

**Results:**

CD patients displayed a larger scatter of individual trials around the average pointing direction (variable error) than normal subjects, whatever the arm used, and the target pointed. When pointing in the left hemispace, all subjects showed a left deviation (constant error) with respect to the target position, which was significantly larger in CD patients than controls, whatever the direction of the abnormal neck torsion could be. Reaction times were larger and peak velocities lower in CD patients than controls.

**Discussion:**

Deficits in the pointing precision of CD patients may arise from a disruption of motor commands related to the sensorimotor imbalance, from a subtle increase in shoulder rigidity or from a reduced agonists activation. Their larger left bias in pointing to left targets could be due to an increased right parietal dominance, independently upon the direction of head roll/jaw rotation which expands the left space representation and/or increases left spatial attention. These deficits may potentially extend to tracking and gazing objects in the left hemispace, leading to reduced skills in spatial-dependent motor and cognitive performance.

## Introduction

Idiopathic Cervical Dystonia (CD) is a movement disorder characterised by prolonged and involuntary contractions of neck muscles and abnormal head posture (Chan et al., 1991; Jankovic et al., 1991). Although its pathophysiology has to be determined yet, recent studies indicate the involvement of the basal ganglia, the cerebellum, the sensorimotor cortex, the parietal cortex, the colliculus, the premotor cortex, as well as of the coupling between these cerebral areas (Naumann et al., 2000; de Vries et al., 2008; Burciu et al., 2017; Filip et al., 2017; Battistella and Simonyan, 2019). Moreover, CD patients show a reduced volume of grey matter in basal ganglia, thalamus, hippocampus, amygdala and a reduced cortical thickness in frontal, parietal, temporal and occipital regions (Tomić et al., 2021). Abnormalities in the different sensory systems have been proposed as predisposing factors to dystonia (Conte et al., 2019).

Whatever the origin of the diseases could be, the neck torsion and lateral bending that may characterise this pathology represent an asymmetry in the sensorimotor neck signals which are fed in the Central Nervous System and that may modify the normal hemispheric balance. In this respect, it has been recently shown that the presence of a trigeminal sensorimotor imbalance is likely leading to an asymmetric Locus Coeruleus activity that may induce cognitive and motor impairments (Tramonti Fantozzi et al., 2019, 2021a, b, c; Grasso et al., 2023). Indeed, CD leads to a movement impairment which goes beyond the neck region: these patients show lower walking speed than normal subjects (Barr et al., 2017) with reduced arm swing (Kägi et al., 2008). Although voluntary arm movements in CD patients have been described as faster/equally long (Katschnig-Winter et al., 2014) with respect to normal subjects, most of the reports indicate that CD is characterised by bradykinesia (Carboncini et al., 2004; Pelosin et al., 2009; Anastasopoulos et al., 2013; Nowak et al., 2013). During reaching movements, the observed directional errors, as defined by the average angular deviation with respect to target location, are increased (Marinelli et al., 2011; Katschnig-Winter et al., 2014).

These motor abnormalities have been related to an anomalous integration of the proprioceptive input, with modification of the internal models of limb dynamics (Pelosin et al., 2009; Marinelli et al., 2011). Several observations are in line with this hypothesis. Indeed, processing of somatosensory input seems to be abnormal within the motor cortex of CD patients (Abbruzzese et al., 2001), while psychophysical data suggest that CD patients have deficits in integrating proprioceptive and vestibular information (Anastasopoulos et al., 1997, 1998). Egocentric body representation, relaying on proprioceptive input, seems to be altered in CD patients (Müller et al., 2005), who favour the use of allocentric reference frames in spatial localization tasks (Ploner et al., 2005). Moreover, it is known that vibratory stimuli applied to the neck muscles, inducing the turned head posture occurring in CD patients, get worse the perception of elbow position, a phenomenon which may impair arm movement control (Tabbert et al., 2022).

This “proprioceptive impairment,” further worsened by the asymmetry introduced in the sensorimotor neck signals, may also lead to deficits in spatial representation. Neck input, in fact, concurs to encode the spatial location, as indicated by the visual target displacement occurring following neck muscle vibration (McIntyre and Seizova-Cajic, 2007) and by the modulation exerted by neck rotation on the responses of parietal neurons to visual stimuli (Brotchie et al., 1995). Studies on spatial localization have shown that CD patients, irrespectively upon the plane and the direction of neck rotation, were characterised by a larger left bias in the line bisection task than normal subjects (Bowers and Heilman, 1980; Chillemi et al., 2018). It has to be pointed out that in normal subjects the left bias in line bisection task is considered an index of the right hemisphere dominance in spatial orientation (Bowers and Heilman, 1980). So, the larger left bias of CD patients would be suggestive of a higher right hemisphere dominance with respect to controls, whatever the direction of neck rotation observed in the patient’s population. This finding can be somehow surprising, since sensory and motor effects of neck rotations are strongly related to its direction (Manzoni et al., 1983; Mergner et al., 1983; Britton et al., 1993; Fitzpatrick et al., 1994).

To the best of our knowledge, no information is currently available in CD patients on pointing movements aimed to far, out-of-reach space, which relays on a different space representation with respect to reaching (Cléry et al., 2015). For this purpose, we studied the kinematic characteristics of pointing, both in normal subjects and in CD patients with the head unrestrained and under normal visual feedback. Pointing precision was investigated by evaluating the mean (constant) directional error between pointing direction and pointed target (Soechting and Flanders, 1989), a parameter which is related to spatial representations and reference frames (Soechting and Flanders, 1989; Vindras et al., 1998, 2016; Khan et al., 2005; Ghafouri and Lestienne, 2006) and allows to highlight possible biases in space representation (Bock, 1986; Soechting and Flanders, 1989; Vindras et al., 1998, 2016; Khan et al., 2005; Ghafouri and Lestienne, 2006; Boyer et al., 2013). Beyond its differences with respect to controls, we aimed to verify whether, in CD patients, the constant directional error was related to the direction and plane of neck rotation, as is the case for the corresponding motor and perceptive effects (Manzoni et al., 1983; Mergner et al., 1983; Britton et al., 1993; Fitzpatrick et al., 1994). Alternatively, it could be independent upon the dystonic neck posture, as it apparently occurs in the line bisection task (Chillemi et al., 2018). A second parameter analysed was the standard deviation of the constant directional error (direction error variability, DEV), which measures the scatter of angular values around the average pointing direction (Soechting and Flanders, 1989). This parameter reflects the precision of the movement and has not been investigated so far in CD patients. In addition, the finger peak velocity (PTV), the reaction (RT) and the movement time (MT) were also evaluated and compared in both control subjects and CD patients.

## Materials and methods

### Subjects

In this study, individuals under follow-up at the Functional Rehabilitation Unit, of the Pisa Hospital, with a clinical diagnosis of idiopathic CD were included. The following criteria were required for inclusion: (1) the presence of a focal CD chronic condition, without spread to other joints, lasting for at least 3 years, (2) lack of botulinum toxin treatment over the preceding 3 months, (3) no head or other body part tremors, (4) no other neurological and psychiatric comorbidities, (5) no recent use of psychotropic drugs. The severity of motor impairment was evaluated by the Toronto Western Spasmodic Torticollis Rating Scale (TWSTRSl; Boyce et al., 2012). Moreover, healthy individuals, who were chosen from the acquaintances of the researchers, served as controls. The absence of mental, neurological, and psychiatric disorders and no history of psychotropic drug use were the requirements for their inclusion in the study.

The experiments were performed in full compliance with the ethical standards laid down in the 1964 Declaration of Helsinki and its later amendments. All subjects signed a written informed consent. All the experimental protocol was approved by the Ethical Committee of the Pisa University (endorsement 15/2020).

### Experimental design

**Figure 1:**
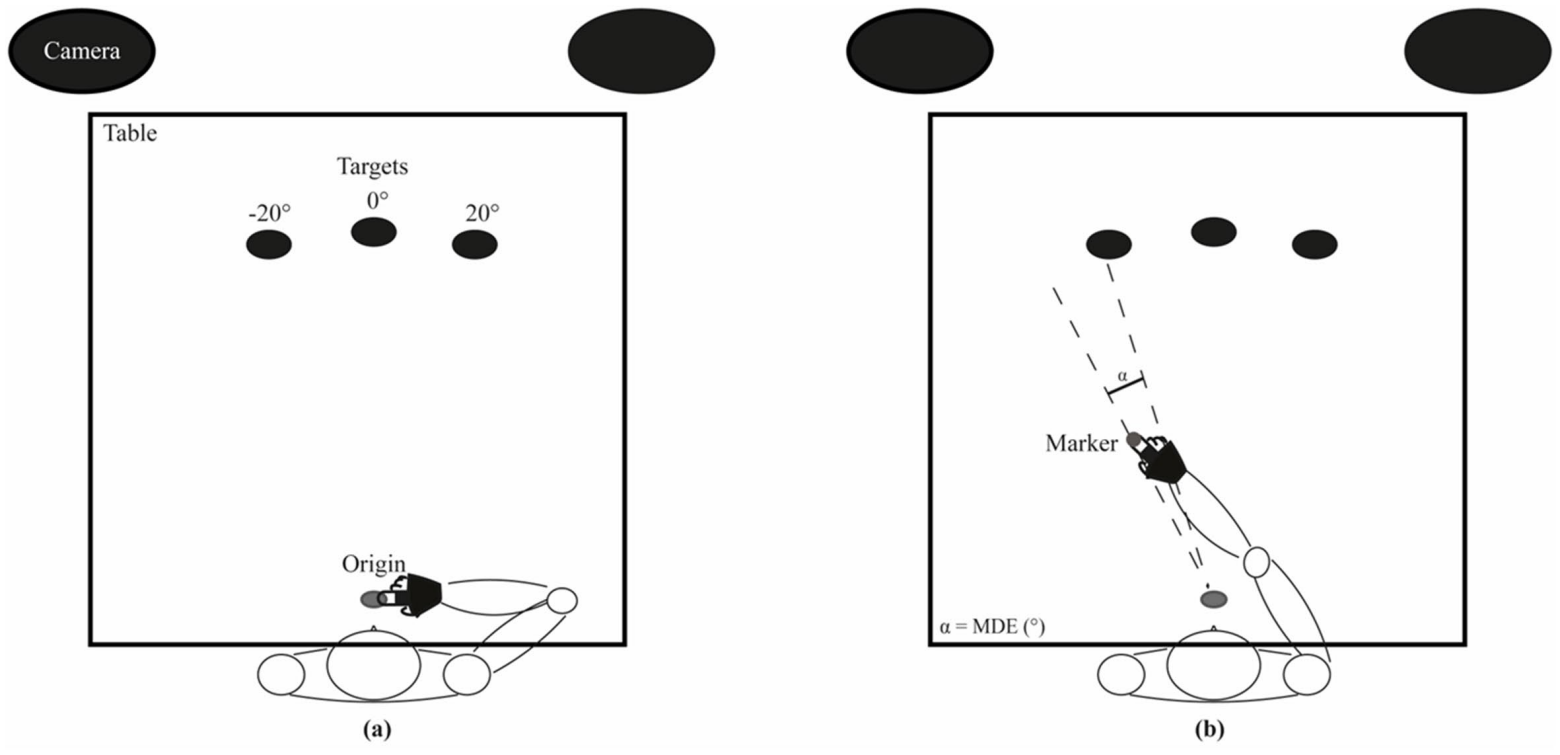
Experimental set-up. **(A)** Resting position: the subject keeps his/her index finger tip at a grey disk located 5 cm in front of the sternum and aligned with the central target. **(B)** Example of a pointing movement to the left target. The angle between the two dashed lines corresponds to the Mean Directional Error (MDE).

The subjects were seated with their right or left arm placed upon a table and were invited to fixate a central target (a 5-mm diameter LED) placed in front of them, at a distance of 1.15 m, according to a standardised protocol (Arrighi et al., 2016). Two additional identical targets were placed 20° left (−20°) and 20° right (+20°) from the midline, at the same distance from the subject (Figure 1). The task was performed at standard artificial lighting, in absence of noise and other disturbing factors. During the task subjects were invited to keep a head position that avoided any discomfort.

The subjects had to keep the tip of their index finger on the movement origin, a thin cork disk glued to the surface of the table, placed 5 cm in front of the subject sternum and aligned with the midline (0°).

Each subject had to perform, with each hand, two blocks of 7 pointing movements towards each target, with full arm extension, as fast as possible. Considering the initial arm position above described, all movements were multi-joint, whatever the target considered (see Figure 1). This number of repetitions was chosen to minimise subject’s fatigue and task disengagement. Participants wore a splint on the pointing hand, blocking the wrist and fixating the index finger in extension and the other fingers in flexion. Across the entire execution of the movement, the hand of the subject was kept in contact with the surface of the table. The GO signal was given by the lighting in a pseudo-random order of one of the 3 LED targets (Figure 1). Subjects were invited to perform a pointing as precise and fast as possible. The target was automatically turned off 4 s following the GO signal.

The movements were monitored using an optoelectronic system (ELITE, BTS, Milano) tracking markers (0.5 cm of diameter) positioned bilaterally on the acromion process, on the lateral epicondyle, on the ulna styloid process and on the tip of the index finger. Images were acquired at 50 Hz by two infrared cameras (Arrighi et al., 2016). The optoelectronic system provided the X, Y and Z coordinates of each marker, as well as their first and second derivative. These data were elaborated with a custom script developed within the software environment MATLAB 7.8.0 (R2009a), computing the kinematic indices described below.

### Statistical analysis

The following variables were evaluated as the average of the 14 movements performed for each arm and target:

a) the Mean (constant) Directional Error (MDE, the angle between the lines connecting the starting point to the target and to the end-position of the finger; Figure 1),
b) the Peak of Tangential Velocity (PTV) of the index finger marker (i.e., the component of the velocity vector tangential to the trajectory of the index finger marker in the horizontal plane),
c) the Movement Time (MT), i.e., the time elapsing between the starting and the end point of the movements, where the velocity raised above and fall below a threshold value set at 3% of PTV (Mutha et al., 2011),
d) the Reaction Time (RT), i.e., the time elapsing between the target lightening and the start of the movement. Finally,
e) the Direction Error Variability (DEV), corresponded to the standard deviation of the 14 values utilised for evaluating the MDE (a).

All these variables were submitted to a 2 Arm (left, right) x 3 Target (−20°, 0°, 20°) repeated measure ANOVA, implemented within the software environment IBM SPSS Statistics v20, with Group (normal subjects, CD patients) as between-subjects factor. The participant’s age was inserted as a covariate within the model. Data are presented as average ± SE. The significance level was set at *p* < 0.05.

No data were available in the literature for estimating *a priori* the sample size allowing a reasonable power for the statistical analysis: indeed, to the best of our knowledge, this was the first study about pointing in CD patients. However, a screening of the literature about reaching, a motor act involving a different space representation, indicated that a sample size of 10–12 CD patients could detect significant differences in reaching precision and movement kinematics with respect to a similar sample of normal controls (Marinelli et al., 2011; Katschnig-Winter et al., 2014). These papers showed effect size values ranging from 1 to 1.89 and evaluations performed with the G*Power software (Düsseldorf University)^1^ indicated that the corresponding total sample size necessary for obtaining a power of at least 0.8 (t-test between independent means, CD patients versus controls) ranged from 10 to 26 subjects. Similar results were obtained from preliminary data of the present study on pointing precision (5 control subjects versus 5 CD patients), indicating an effect size of 1.09 and a total sample size of 22 individuals for reaching the 0.8 power criterion. Power estimations could be also performed ex post for supporting the reliability of the observed differences between CD patients and control participants (see Results section). No particular treatment for missing points had to be applied, due to availability of complete data acquisition for each subject.

The database of the present study is available.^2^

## Results

### Subjects

The 18 CD patients (mean age: 51.65 ± 11.02, SD, years, 10 females) and the 11 healthy controls (mean age: 50.67 ± 8.95 years, 7 females) enrolled in this study were right-handed, as confirmed by their scores on the 10-item version of Edinburgh Handedness Inventory (Oldfield, 1971). Observation of the spontaneously maintained head posture revealed that most of the CD patients (*n* = 14) had a prevalent pattern of horizontal neck twist (torticollis; Chillemi et al., 2018, left head rotation: *n* = 8, right head rotation: *n* = 6) and four of them a prevalent pattern of (right) neck sideways bending (laterocollis; Chillemi et al., 2018). In general, manual evaluation revealed a contralateral sternocleidomastoid hypertonus in patients with prevalent rotatocollis, while an ipsilateral trapezius hypertonus in prevalent laterocollis patients. It must be pointed out, however, that manual examination, a coarse way to assess muscle tone, was limited to these superficial muscles, while a given head posture arises also from the contribution of more deeply located muscles.

In CD patients, the resistance to passive limb rotation was normal at the elbow but looked higher than normal at the shoulder joint. Data relative to the patterns of abnormal neck postures and additional patients’ demographic and clinical information are provided in Table 1. No pain or discomfort was experienced by CD patients during all the experimental session.

**Table 1:** Clinical features of dystonic patients.

Cases	Sex	Age (years)	Duration (years)	Type of CD	TWSTRS			
					Severity score	Disability score	Pain score	Total score
1	F	48	5	Torticollis (L)	8.00	10.00	6.50	24.50
2	M	67	20	Torticollis (L)	16.00	11.00	12.75	39.75
3	M	42	6	Torticollis (L)	6.00	12.00	9.75	27.75
4	F	51	2	Laterocollis (R)	11.00	9.00	6.00	26.00
5	F	68	8	Torticollis (R)	23.00	23.00	15.00	61.00
6	M	54	6	Torticollis (L)	19.00	13.00	13.50	45.50
7	M	32	14	Laterocollis (R)	20.00	14.00	9.00	43.00
8	F	65	14	Torticollis (L)	11.00	14.00	9.50	34.50
9	F	49	3	Torticollis (L)	8.00	11.00	4.25	23.25
10	F	53	21	Torticollis (L)	13.00	16.00	1.50	30.50
11	M	74	21	Torticollis (R)	19.00	27.00	15.00	61.00
12	M	55	3	Torticollis (R)	21.00	27.00	18.00	66.00
13	F	47	6	Torticollis (R)	20.00	22.00	16.00	58.00
14	F	48	21	Laterocollis (R)	27.00	22.00	0.00	49.00
15	F	65	45	Laterocollis (R)	18.00	19.00	12.25	49.25
16	M	45	16	Torticollis (R)	22.00	24.00	16.25	62.25
17	M	44	10	Torticollis (L)	9.00	11.00	7.50	27.50
18	M	41	5	Torticollis (R)	18.00	11.00	14.75	43.75

Scores relative to the Toronto Western Spasmodic Torticollis Rating Scale (TWSTRS; Boyce et al., 2012) have been reported.

### Mean directional error and direction error variability

The Mean (constant) Directional Error (MDE) illustrated in Figure 1 depended significantly upon the arm used [Arm effect: *F*(1,26) = 8.657, *p* = 0.007], the target pointed [Target effect: *F*(2,52) = 10.928, *p* = 0.001], and their combination [Arm × Target interaction: F(2,52) = 3.429, *p* = 0.040], in both control subjects and CD patients.

As to the Arm effect, average MDE values across different targets were displaced to the left (−3.82 ± 0.77°) and to the right (1.27 ± 0.82°) when pointing with the left and right arm, respectively.

As to the Target effect, subject’s pointing tended to be displaced to the left for the left target (−6.93 ± 1.04°) and to the right for the right target (4.33 ± 0.82°) whatever the pointing arm, while a small error bias occurred for the central target (−1.22 ± 0.59°). All comparisons between targets were highly significant (*post-hoc*, *p* < 0.0005).

Finally, the Arm x Target effect was attributable to the difference in MDE observed between left and right arm pointing to the left (left arm: −10.18 ± 1.18°, right arm: −3.69 ± 1.21°, *p* < 0.0005) and to the right (left arm: 1.28 ± 0.95°, right arm: 7.38 ± 1.05°, *p* < 0.0005) targets, but not to the central one (left arm: −2.57 ± 0.78°, right arm: 0.12 ± 1.00°, *p* = 0.056).

**Figure 2:**
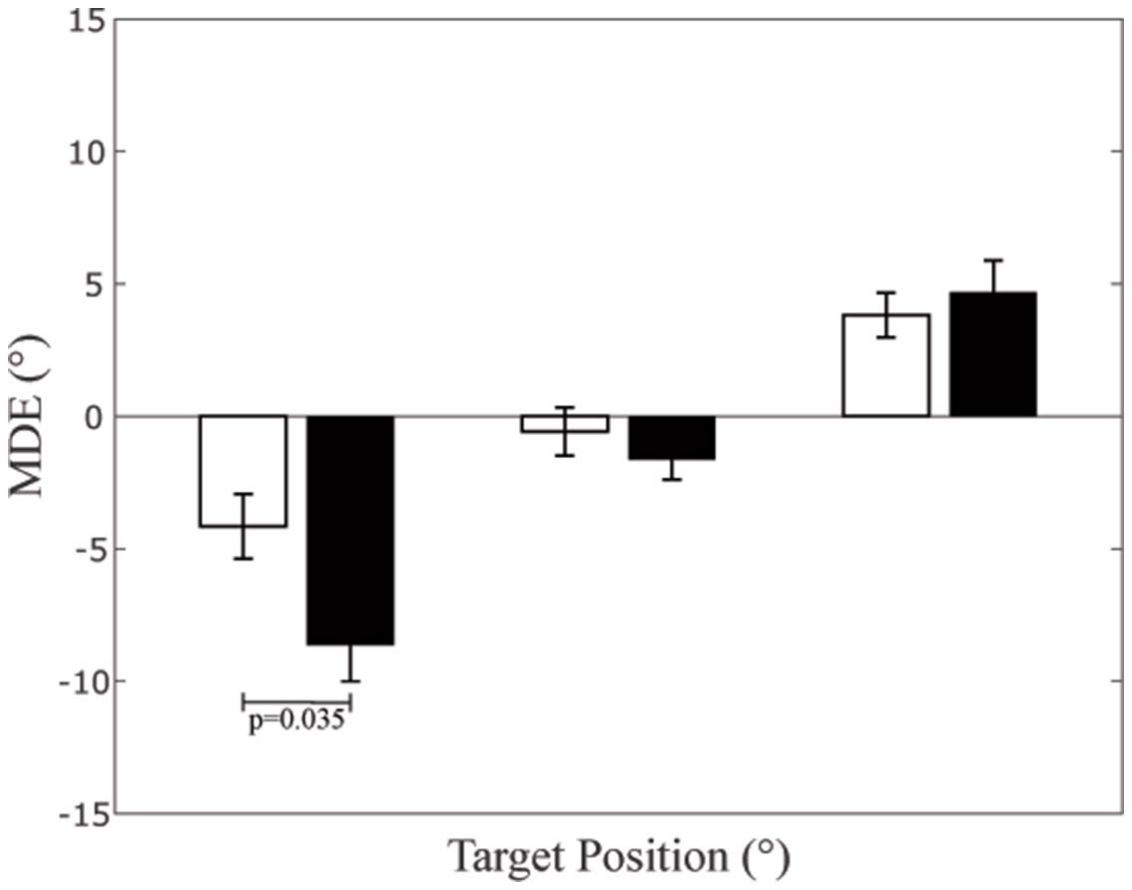
Mean Directional Error (MDE) at different target positions. The bar height represents the average MDE values, evaluated in each subject as the mean value of left and right arm. The dispersion bars correspond to the SE. Data relative to CD patients and controls have been separately represented by black and white columns, respectively.

Differences between control subjects and CD patients are described in Table 2. When the between-subjects factor was considered, a significant Target x Group (normal subjects, CD patients) effect was observed [F(2,52) = 4.655, *p* = 0.024]. As shown in Figure 2, when pointing leftwards, dystonic subjects showed a pointing error to the left (MDE: −8.63 ± 1.38°, average of left and right arms) significantly larger with respect to controls (−4.16 ± 1.22°, *p* = 0.035). Although the significance level of this comparison was just below the 0.05 level, an ex-post power evaluation for a t-test between independent means based on the corresponding effect size (1.37) returned a very high power value (0.975), thus reinforcing the reliability of the result. No significant differences could be observed for the remaining two targets (0°: *p* = 0.404; +20°: *p* = 0.626).

**Table 2:** Average mean directional error (MDE) and direction error variability (DEV) values in CD patients and control subjects.

	Dystonic patients (*n* = 18)	Controls (*n* = 11)
*Left arm*		
MDE (°)		
Target −20°	−12.5 ± 1.3	−5.7 ± 1.3
Target 0°	−3.3 ± 0.9	−0.5 ± 0.7
Target 20°	1.5 ± 1.2	1.5 ± 0.7
DEV (°)		
Target −20°	3.5 ± 0.4	1.7 ± 0.3
Target 0°	2.3 ± 0.3	1.3 ± 0.2
Target 20°	3.7 ± 0.5	1.3 ± 0.2
*Right arm*		
MDE (°)		
Target −20°	−4.7 ± 1.5	−1.6 ± 1.5
Target 0°	0.1 ± 1.3	0.5 ± 0.9
Target 20°	7.8 ± 1.3	6.8 ± 1.2
DEV (°)		
Target −20°	3.5 ± 0.7	1.5 ± 0.2
Target 0°	2.6 ± 0.5	1.7 ± 0.3
Target 20°	2.7 ± 0.7	1.9 ± 0.4

Values relative to different targets and arms are given. Dispersion values represent SE. See text for further explanations.

All patients, when pointing to the left target, showed the same left bias, irrespectively upon the direction and the plane of neck rotation. More specifically, the average MDE values obtained in patients with left and right torticollis corresponded to −8.94 ± 2.69° (*n* = 8) and to −8.81 ± 1.85° (*n* = 6), respectively (*p* = 0.972). Moreover, these values were not significantly different than that observed for subjects with (right) laterocollis (−7.73 ± 2.44°; left rotatocollis: *p* = 0.781, right rotatocollis: *p* = 0.729).

In principle, the left MDE bias could also depend upon the side of neck hypertonus (contralateral to neck deviation in torticollis, while ipsilateral in laterocollis). Grouping patients according to the hypertonus side indicated that left (*n* = 6) and right (*n* = 12) neck hypertonus was associated with similar MDE values (left hypertonus: −8.82 ± 1.84°, right hypertonus: −8.54 ± 1.91°, *p* = 0.928).

The values of the Direction Error Variability (DEV), which measures the scatter around the average pointing direction, were not dependent upon the arm used for pointing, upon the pointed target (20° left, centre and 20° right) or upon the combination of the two factors, as no significant Arm, Target or Arm x Target effects could be found when DEV was analysed by ANOVA. However, a significant Group effect (CD patients, controls) was observed [*F*(1,26) = 7.177, *p* = 0.013], without any interaction with Arm or Target. The average DEV was larger in CD patients (3.17 ± 0.35, SE,°) than in normal controls (1.59 ± 0.21°).

**Figure 3:**
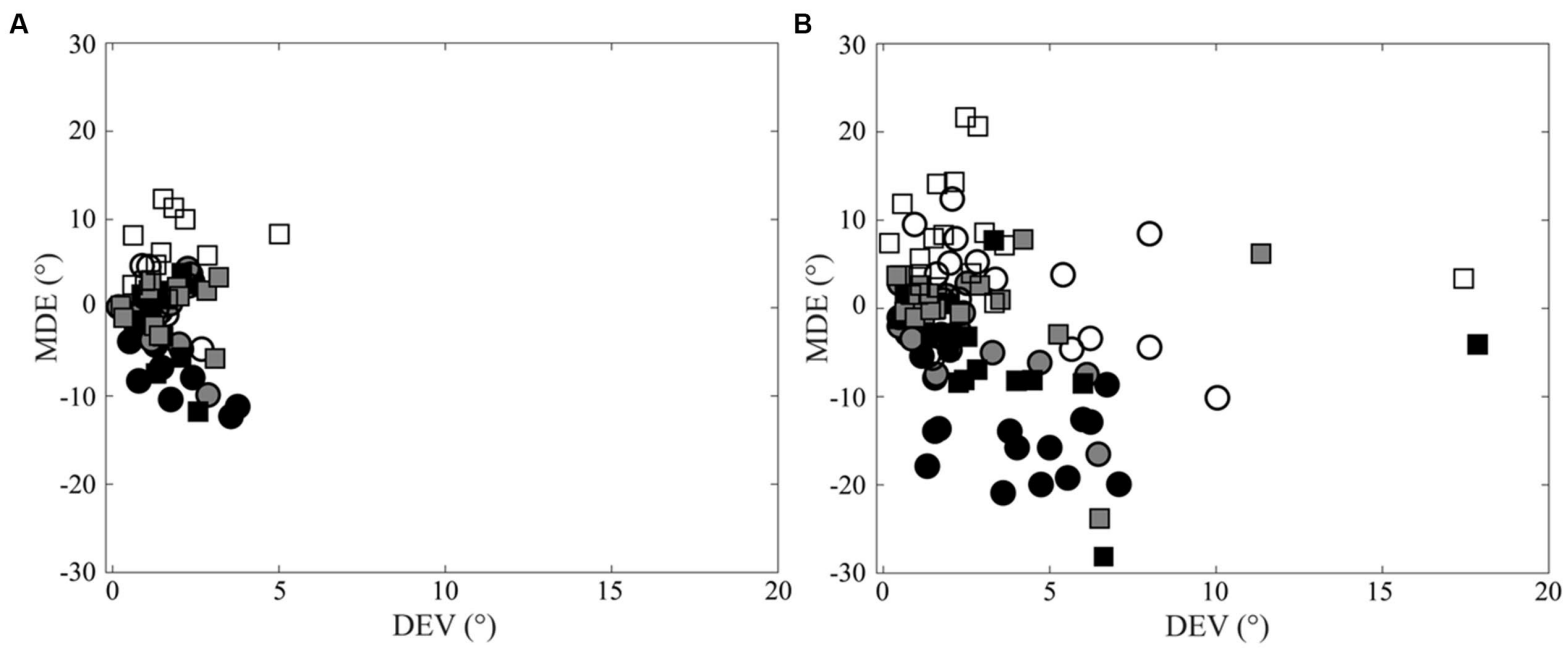
Scatterplots of Direction Error Variability (DEV) versus Mean Directional Error (MDE). **(A)** Control subjects. **(B)** CD patients. Right and left arm data are indicated by square and circles, respectively. Black, grey and white filling of the symbols indicates −20°, 0° and 20° targets, respectively.

Figure 3 summarises and highlights the difference in the pointing precision for normal subjects (Panel a) and CD patients (Panel b) by showing the scatterplots of DEV and MDE for the different targets and arms.

### Dystonia severity and mean directional error

Dystonia was more severe in patients with rightward than in those with leftward torticollis (TWSTRS severity score- right: 20.50 ± 0.76, left: 11.25 ± 1.58; *p* < 0.0005). Despite this difference in the severity of dystonia, comparable left deviations were observed when pointing to the left target in both groups. Consistently, dystonia severity was significantly larger in patients with left than in those with right neck hypertonus (TWSTRS severity score - right: 20.50 ± 0.76; left: 13.83 ± 1.8 *p* = 0.004). Yet, the two groups of patients showed the same level of MDE bias when pointing to the left target. Consistently, no significant correlation was found between dystonia severity and extent of pointing deviation.

### Peak of tangential velocity

**Figure 4:**
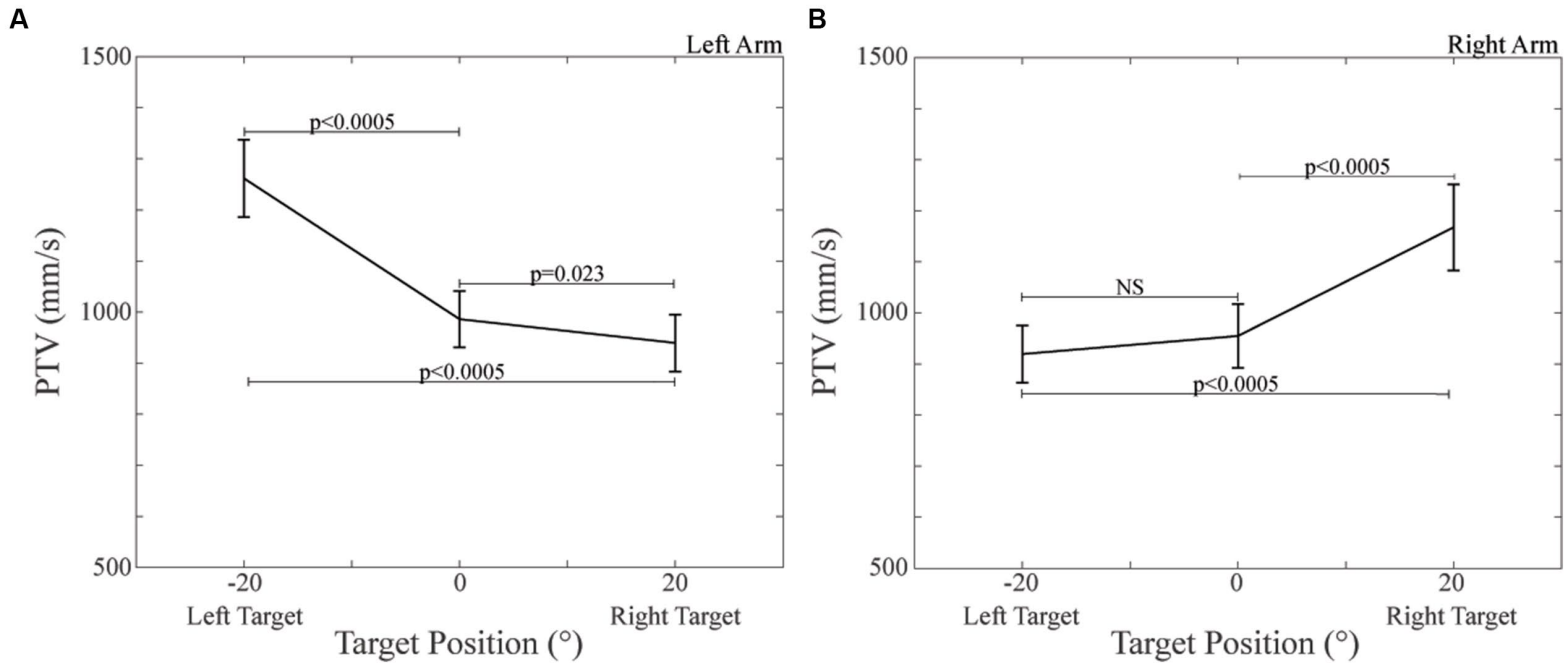
Peak of Tangential Velocity (PTV) values observed during pointing to the different targets. **(A)** PTV, left arm pointing. **(B)** PTV, right arm pointing. The dispersion bars correspond to the SE.

The ANOVA model applied to PTV disclosed a significant Arm × Target effect [*F*(2,52) = 7.198, *p* = 0.006]. As shown in Figure 4, when pointing movements were performed with the left arm, subjects reached the highest velocity at the left target (−20°: 1261.81 ± 75.95 mm/s); velocity decreased when pointing at the centre (0°: 986.09 ± 55.32 mm/s, *p* < 0.0005) and at the right target (+20°: 939.57 ± 57.75 mm/s, *p* < 0.0005). Also the difference in velocity between centre and right targets was significant (*p* = 0.023). A specular behaviour was observed for the right arm, as velocity was the highest when pointing at the +20° target (1202.50 ± 84.45 mm/s) and progressively decreased at the centre (989.85 ± 62.63 mm/s, *p* < 0.0005) and at the left target (954.46 ± 55.97 mm/s, *p* < 0.0005). The difference in velocity between centre and left targets was not significant (*p* = 0.136). The difference in the PTV values between the two arms was significant when pointing at the left and right (both *p* < 0.0005), but not at the centre target (*p* = 0.902).

As indicated by a significant Group effect [F(1,26) = 4.332, *p* = 0.047] the overall velocity of control subjects (1208.56 ± 113.25 mm/s) was higher than that of CD patients (962.31 ± 64.91 mm/s).

### Movement time and reaction time

When ANOVA was applied to MT values no significant Arm, Target, Group effect and interactions could be observed.

Reliable RT data could be obtained in 7 control subjects and in 15 CD patients. Repeated measures ANOVA revealed a significant Group [*F*(1,19) = 10.556, *p* = 0.004] effect. Indeed, the RT value was higher for CD patients (740.63 ± 43.62 ms) with respect to control subjects (497.61 ± 27.52 ms).

### Correlations between variables

**Figure 5:**
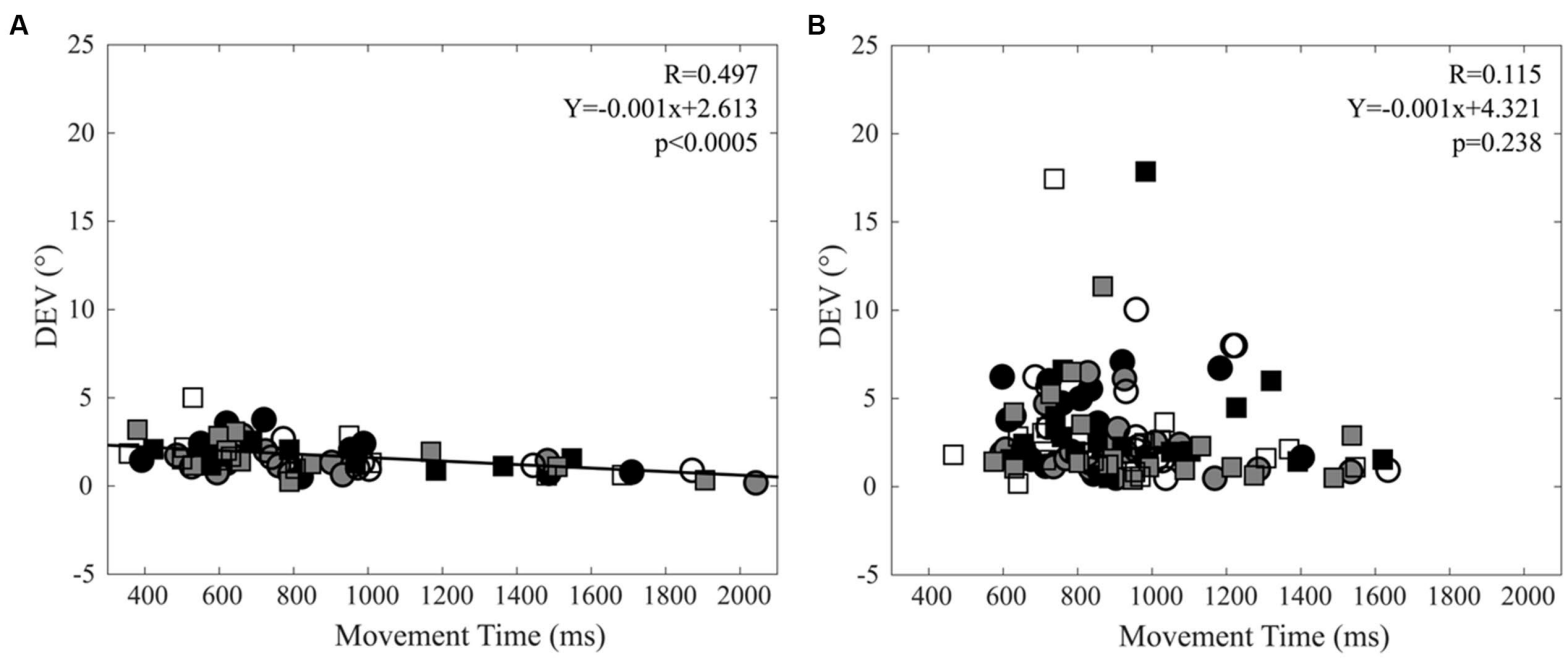
Relation between Direction Error Variability (DEV) and Movement Time (MT). **(A)** Control subjects. **(B)** CD patients. Right and left arm data are indicated by square and circles, respectively. Black, grey and white filling of the symbols indicates −20°, 0° and 20° targets, respectively. The regression line and the relative data refer to all the plotted points.

When data relative to different targets were pooled, a significant correlation could be observed for both the right (*R* = 0.516, *Y* = −0.001X + 2.737, *p* = 0.003) and left (*R* = 0.478, Y = −0.001X + 2.312, *p* = 0.007) arm between variable error (DEV) and MT in control subjects, but not in CD patients. These data are summarised in Figure 5, where left and right arm values pointing at the three different targets - plotted with different symbols - have been analysed together.

MDE was independent of MT for both arms and groups and the same held true when the absolute MDE values were considered. Moreover, neither MDE, nor DEV were correlated to the RT, whatever group was considered.

**Figure 6:**
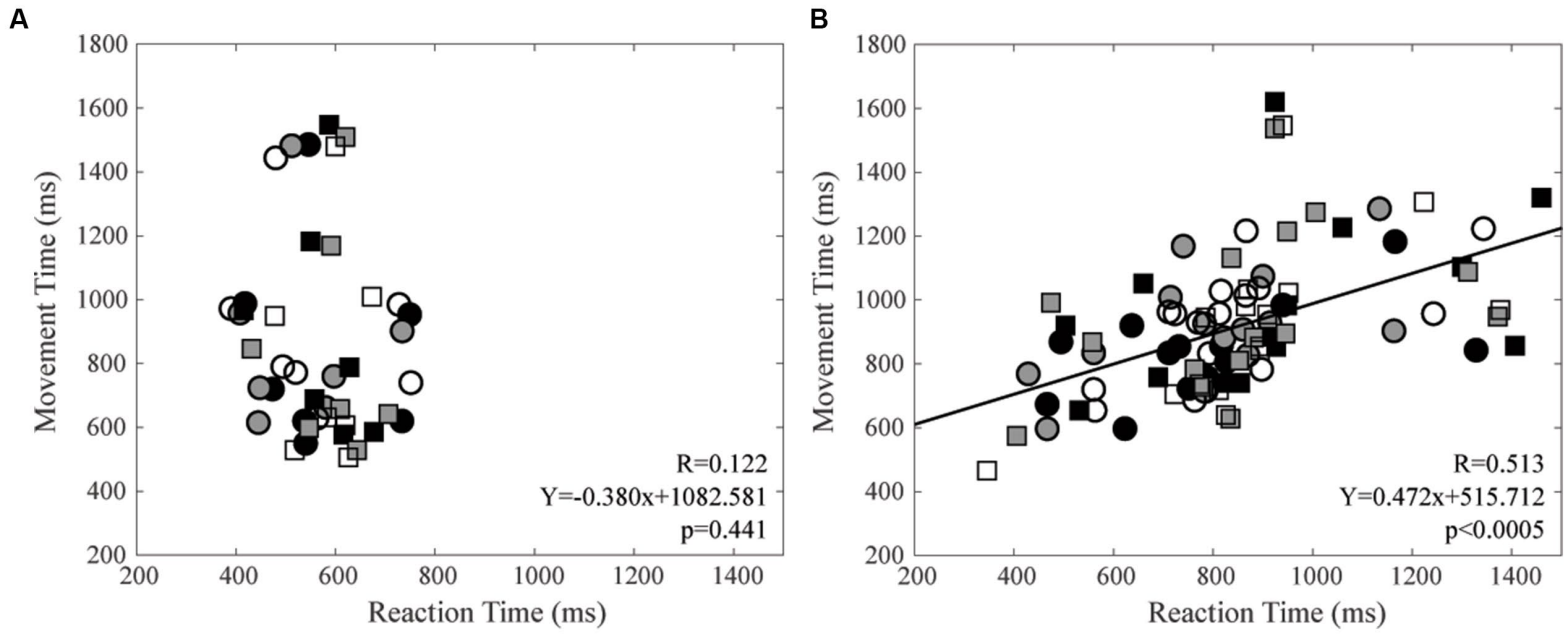
Relation between Movement Time (MT) and Reaction Time (RT). **(A)** Control subjects. **(B)** CD patients. Right and left arm data are indicated by square and circles, respectively. Black, grey, and white filling of the symbols indicates −20°, 0° and 20° targets, respectively. The regression line and the relative data refer to all the plotted points.

Finally, MT and RT were not correlated among control subjects, while this was the case among CD patients, for both right (*R* = 0.474, Y = 0.476X + 523.79, *p* = 0.001) and left (*R* = 0.563, Y = 0.446X + 525.28, *p* < 0.0005) arms. These data are summarised in Figure 6, where data relative to the right and left arm pointing at the three different targets, plotted with different symbols, have been analysed together.

### Relation between clinical and behavioural variables

With respect to control participants, CD patients showed a larger (left) bias in pointing to the left target with both arms. Moreover, whatever the target and the arm, they displayed higher pointing variability and reaction time, as well as lower peak tangential velocity. The possible correlation between these parameters and the TWSTRS score, as well as with its relative subscales (severity, disability and pain) was investigated by linear regression analysis. No significant correlations could be found, whatever the behavioural parameter and the TWSTRS score/subscale could be.

## Discussion

Pointing movement towards out of reach targets are well suited for investigating the (neural) representation of the far space, which is different from that utilised for performing reaching movements (Cléry et al., 2015). Information about far space coding can be captured by evaluating the mean (constant) directional error between pointing direction and pointed target (Soechting and Flanders, 1989). Indeed, this parameter has revealed biases in space representation of normal subjects (Bock, 1986; Vindras et al., 1998, 2016; Khan et al., 2005; Boyer et al., 2013) and may be therefore utilised for the same purpose in CD patients. When normal subjects pointed towards out-of-reach space with both hands, their aiming directions to left and right-located targets were displaced to the left and to the right, respectively, as indicated by the corresponding MDE values (Figure 2). It is unlikely that this finding is related to mechanical constraints, since left and right arms, despite showing the same directional error, underwent opposite mechanical constraints when rotating in each direction of the horizontal plane (any of the two arms moved easier away from the trunk than towards).

Similar results have been observed while pointing to visual targets, with the head restrained (Bock, 1986), and to acoustic targets when the head is free (in blindfolded subjects; Boyer et al., 2013). All these findings can be interpreted according to the same perspective of spatial localization error, as an overestimation of the eccentricity of both visual and acoustic targets, making the perceived space wider than the real one (Bock, 1986; Boyer et al., 2013).

CD patients, when compared to normal subjects, showed larger deviations to the left when pointing to the left target. Although the significance level (*p* = 0.035) of this result is just below 0.05, its power is very high (almost one) and supports its reliability. The larger pointing bias in CD patients suggested by the present study, cannot arise from a proprioceptive disruption related to the abnormal neck input (Tabbert et al., 2022), since it was observed only for a specific pointing direction (left targets), while a difficulty in arm control should be evident in all the sectors of the workspace. Moreover it was independent upon the dystonia severity, as well as upon the direction of head rotation/neck hypertonus. This is at variance with what observed in normal subjects, in whom an asymmetric neck input elicits effects which depend upon the direction of head rotation both in the motor (Manzoni et al., 1983; Britton et al., 1993; Fitzpatrick et al., 1994) and in the perceptual domain (Mergner et al., 1983; Fitzpatrick et al., 1994). Overall, these findings indicate that the left-expanded pointing of CD patients are not due to an abnormal proprioceptive control of the movement, but point towards a higher level dysfunction in spatial processing and localization.

Our data retrace the results of line bisection experiments, showing that the larger left bias of CD patients was larger than controls (Bowers and Heilman, 1980; Chillemi et al., 2018), whatever the direction of neck rotation might have been. Although peripersonal, reaching space and far space (where pointing is oriented) are coded by different neural representations (Cléry et al., 2015, 20), a similar mechanism could be involved in both studies. In normal right-handers, the left-oriented bias in line bisection task is attributed to the spatial dominance of the right hemisphere (Bowers and Heilman, 1980; Brown and Kosslyn, 1993; Dieterich and Brandt, 2018). Following right hemispheric stroke, which elicits the human hemineglect syndrome (Karnath et al., 1993; Kerkhoff, 2001; inability to orient movements and attention towards the left hemispace) the line bisection task becomes strongly biased to the right side (Kerkhoff, 2001). For these reasons, the larger left bias of CD patients in line bisection task has been explained by an increased dominance of their right over left hemisphere (Chillemi et al., 2018). It is possible that also the left bias observed in CD patients (with left or right head turning) during pointing is the expression of a slight increase in right hemisphere dominance, possibly associated to an enhancement of visual attention to the left space. As illustrated in Supplementary Figure S1, this could be the case provided that:

1. CD patients show a bilateral increase in muscle tone, whose extent is larger than the difference observed between the two sides,
2. The neck input has a dominant representation in the right hemisphere.

Indeed, although CD patients show a net head torque in the same direction of the torticollis, the resistance to head displacement is significantly increased for both directions of head rotation, thus indicating that, apart from the asymmetry, there is an abnormal, bilateral increase in the neck muscle tone (Anastasopoulos et al., 2014). As to the neck input to cerebral cortex, it is known that a right side neck vibration elicits a bilateral increase of blood-oxygen-level-dependent (BOLD) signal in areas 3a and 2 of the primary somatosensory cortex, in the secondary somatosensory cortex, in the parieto-insular vestibular cortex, and in the intraparietal sulcus (Fasold et al., 2008), while vibration of left neck muscles activates the insula and the second somatosensory area selectively on the right side (Bottini et al., 2001). Overall, these data suggest that the neck input has indeed a dominant representation in the right hemisphere. In addition, neck signals diffusely converge with vestibular signals at the cortical level (Mergner et al., 1985; Shinder and Newlands, 2014), and both inputs contribute to self-motion perception (Mergner et al., 1991). It is known that the vestibular input has a dominant representation within the right (spatially dominant) hemisphere of right-handers (Dieterich et al., 2003), making likely a similar distribution also for the neck sensory input.

Within this framework, a bilateral increase in tonic neck input would enhance the activity of the right more than the left parietal networks. This would reinforce the dominance of the right over the left hemisphere in CD patients, slightly expanding the left space representation and/or shifting the attentional focus to the left side, whatever the localization of neck hypertonus could be (Supplementary Figure S1).

It is of interest that the line bisection task shows a rightwards bias in chronic vestibular hypofunction, whatever the lesioned labyrinth (right or left) might be, suggesting a right hemispheric deficits in these subjects (Saj et al., 2020). Abnormal vestibular input resulting from head deviation with respect to gravity in CD patients may concur to left expansion of CD patients far space which is indicated by the present experiments.

Further investigations in CD patients are necessary to assess whether the left bias in space representation observed during pointing can be generalised to sensorimotor spatial performance other than reaching and pointing. Moreover, whether or not this phenomenon can be observed also in left-handed patients has still to be determined. In fact, in our sample, only right-handed patients were recruited and analysed.

The data of the present study indicate that, irrespectively of the pointing arm and of the pointed targets, CD patients showed a larger scatter of their final finger positions (DEV) with respect to the average pointing direction. The larger DEV values of CD patients could be explained either by higher levels of “planning” or “execution” noise (van Beers, 2009). The “execution” noise can be attributed to a low firing rate of motoneurons, leading to unfused twitches and tension fluctuations. These fluctuations can be enhanced by an increased degree of synchronisation and variability in motoneurons discharge, as well as by the intrinsic variability of the twitches (Faisal et al., 2008). It has been shown that, in CD patients without segmental spread of dystonia, arm movements in the horizontal plane are associated to a lower amplitude of electromyographic (EMG) agonist burst with respect to normal controls (Carboncini et al., 2004), a finding consistent with a reduced firing frequency of recruited motor units. No data concerning motor unit synchronisation and discharge regularity, as well as twitches variability during arm movement, are available for these patients. The “planning” noise is due to the variability in preparatory motor cortical activity which affects the characteristics of the ensuing movement (Churchland et al., 2006a, b). The possibility that “planning” noise affects the pointing variability in CD patients cannot be excluded, although the present study does not allow to draw any final conclusion. It must be pointed out that the abnormal head posture assumed by these patients can be considered as a sensorimotor imbalance in the neural systems controlling head posture. In normal subjects, the presence of a sensorimotor trigeminal imbalance leads to an enhanced activation of cerebellar and cortical motor areas during finger movements, consistent with the hypothesis of an enhancement of “planning” noise (Tramonti Fantozzi et al., 2019). It is possible, therefore, that a similar phenomenon occurs in CD patients, making movement planning and execution more difficult. Finally it has to be mentioned that an altered neck proprioceptive input may get worse the limb position sense (Tabbert et al., 2022), possibly leading to a movement impairment which could account for the increase in DEV observed in CD patients.

In the present study, PTV decreased when moving with a given arm from the ipsilateral to the contralateral target, as it could be expected, due to the higher mechanical constraint affecting contralateral pointing movements. Consistently with previous investigations, its value was lower in CD patients with respect to normal participants, thus pointing to bradykinesia as a characteristic of this specific disorder (Carboncini et al., 2004; Pelosin et al., 2009; Anastasopoulos et al., 2013; Nowak et al., 2013), in line with what it is generally observed for dystonic disturbances (Paparella et al., 2023). This finding is consistent with the reduced agonist burst observed by Carboncini et al. (2004). Despite that, the movement time of CD patients was only slightly larger than that of control participants. This could be due to the occurrence of final adjustments of pointing direction that prolonged the MT duration, blurring the differences between the two groups.

In control subjects, a negative correlation was observed between DEV and MT in agreement with the well-known trade-off between velocity and precision (Fitts, 1954; Wright and Meyer, 1983; Harris and Wolpert, 2006). These correlations were lost in CD patients, owing to the larger scatter of DEV values observed in this group (Figure 5).

RT values were significantly larger in CD patients than in controls. Previous investigations showed that reaction times to target enlightening were similar in the two groups (Pelosin et al., 2009), when subjects performed a reaching task based on a hand-controlled displacement of a target on a computer screen. Shorter reaction times of controls with respect to CD patients was instead observed during larger reaching movements towards visual targets (Porcacchia et al., 2014). It is possible that differences in RT between controls and CD patients can be observed only when movement amplitude is rather large. This hypothesis would be consistent with the results of our study, where the arm reached full extension, leading to larger displacement and RT values with respect to previous studies.

An increase in RT could be either of central or peripheral origin. An increase in central delay could occur in CD patients, due to a reduced attention to sensory stimuli and/or to a worsening of neural processing, i.e., to a cognitive impairment elicited by the hemispheric imbalance associated with the abnormal head posture. It has been shown, indeed, that an hemispheric imbalance elicited by an asymmetric trigeminal input leads to a reduced visuospatial performance (Tramonti Fantozzi et al., 2021a, b). In the present experiments the reaction time is evaluated as the delay between the target illumination and the movement beginning, the latter corresponding to the time when the arm velocity reaches 3% of the maximal PTV. For this reason, the larger RT value in CD patients may also depend upon a longer time needed to convert motoneuronal discharge into muscle contraction, due to the increased proximal stiffness and reduced agonist burst (Carboncini et al., 2004). Both factors might have delayed the initial arm displacement (necessary for detecting the beginning of the movement), prolonging the estimated RT value. Data shown in Figure 6 suggest that factors prolonging the RT in CD patients occurred mainly when the subjects programmed long duration movements. Further experiments with a large sample size of control subjects are necessary to verify the reliability of these speculations.

We have to acknowledge that this study has some limitations. First, the evaluation of the dystonia pattern was performed by direct observation of the patient’s neck posture and by direct palpation of the sternocleidomastoid and trapezius muscles. This approach may only assess the predominant pattern of dystonia and does not pick up the contribution of deep neck muscles that concur to determine head position. Moreover both torticollis and laterocollis can be present, although to different extent, in the same subject. Second, the sample of CD patients was larger than that of controls subjects. Third, we compared CD patients and control subjects only in their resting condition, where CD patients, but not control subjects, showed a strong asymmetry in proprioceptive neck input. Comparison of CD patients with control subjects keeping rotated head postures as a consequence of neck vibration (Tabbert et al., 2022) would have allowed to disentangle between-group differences related to chronic brain changes, rather than to the resting neck posture. This problem has to be addressed by further experiments and may clarified relevant pathophysiological aspects in CD. Finally, the significance of the difference in bias when pointing to the left target was just below 0.05. Although this result was reinforced by a very high-power value, further experiments with a larger number of subjects may be appropriate for achieving a higher significance level. Despite these limitations, to the best of our knowledge, the present study has investigated for the first time the dynamics of pointing in CD patients, giving evidence for interesting differences with respect to normal controls.

## Data availability statement

The datasets presented in this study can be found in online repositories. The names of the repository/repositories and accession number(s) can be found at: https://osf.io/hb75e/.

## Ethics statement

The studies involving humans were approved by Ethic Committee for Human Research of the Pisa University. The studies were conducted in accordance with the local legislation and institutional requirements. The participants provided their written informed consent to participate in this study.

## Author contributions

MPTF: Conceptualization, Formal analysis, Writing – original draft, Writing – review & editing, Data curation, Validation, Visualization. RB: Formal analysis, Writing – review & editing, Data curation, Investigation, Methodology. AC: Investigation, Methodology, Writing – review & editing. LB (Fourth author): Investigation, Methodology, Data curation, Writing – review & editing. PA (Fifth author): Conceptualization, Data curation, Methodology, Software, Supervision, Validation, Writing – review & editing. PA (Sixth author): Conceptualization, Data curation, Methodology, Software, Supervision, Validation, Writing – review & editing. LB (Seventh author): Methodology, Resources, Writing – review & editing. MCC: Project administration, Resources, Supervision, Writing – review & editing. LB (Ninth author): Funding acquisition, Resources, Writing – review & editing. PB: Methodology, Resources, Writing – review & editing. UF: Conceptualization, Supervision, Validation, Writing – review & editing. DM: Conceptualization, Data curation, Formal analysis, Project administration, Supervision, Writing – original draft, Writing – review & editing.

## Footnotes

^1^ https://www.psychologie.hhu.de/arbeitsgruppen/allgemeine-psychologie-und-arbeitspsychologie/gpower

^2^ https://osf.io/hb75e/
