# Supplementary material for: Pointing in cervical dystonia patients
Source: Front Syst Neurosci. 2023 Nov 28;17:1306387. doi: 10.3389/fnsys.2023.1306387 (PMC10714009; doi:10.3389/fnsys.2023.1306387)
Supplement: Supplementary file 1 [file Data_Sheet_1.PDF]

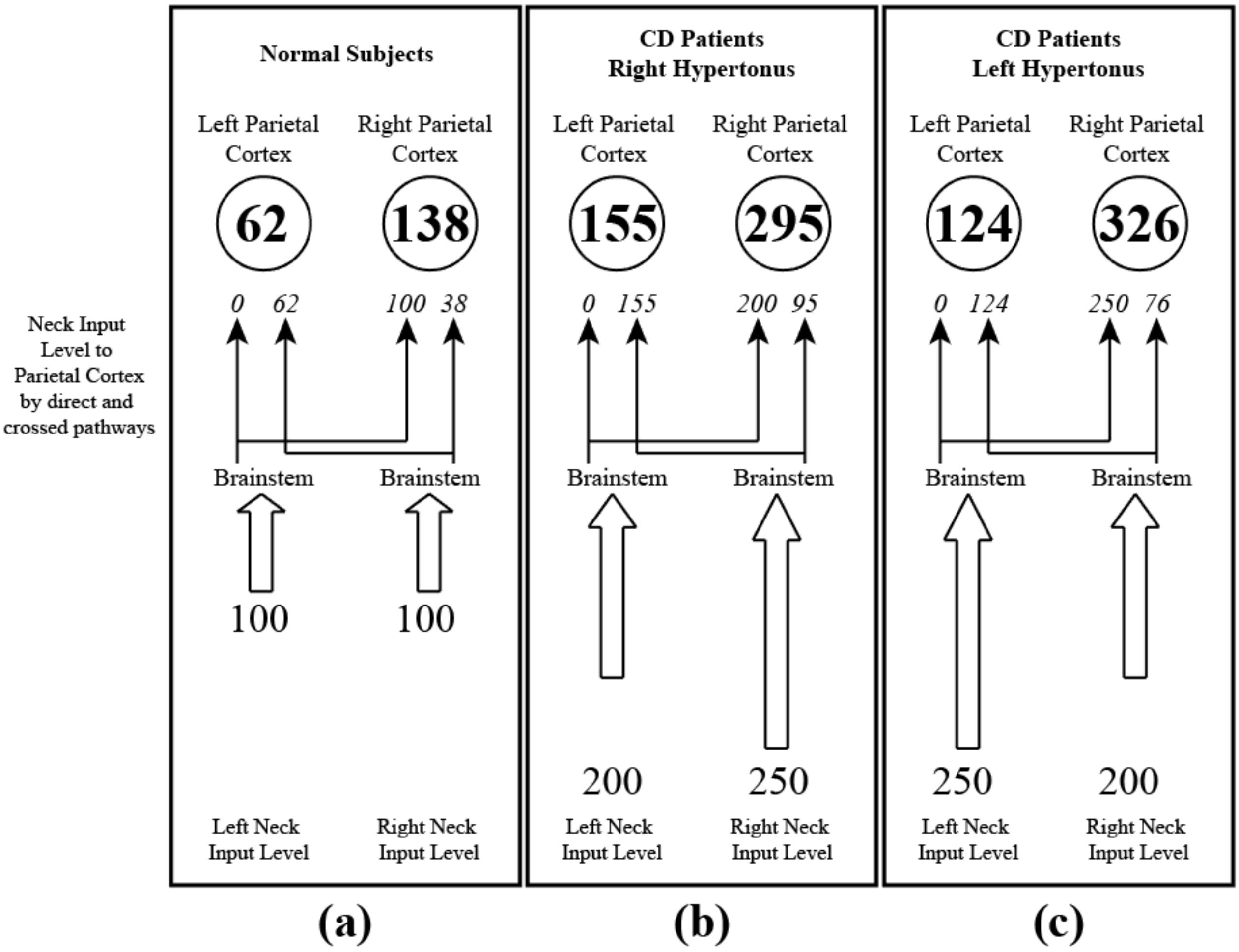

Supplementary Figure S1.

Modelling neck input level to parietal cortex. Numbers: level of activity in the direct and crossed neck pathways to parietal cortex. The exclusively contralateral projection of the left side neck input has been inferred from Bottini et al., 2001, while the bilateral projection of the right side neck input from Fasold et al., 2008. Moreover, the percentages of ipsilateral and contralateral projections from right side neck afferents refer to the relative extent of the parietal clusters activated by neck vibration (see Table 2 from Fasold et al., 2008). Circled numbers refer to the overall neck input to the left and right parietal cortices in normal subjects (a) and in CD patients with right (b) or left (c) neck hypertonus.
